# Second Primary Malignancies and Myeloma Therapy: Fad or Fact?

**DOI:** 10.18632/oncotarget.661

**Published:** 2012-09-15

**Authors:** Saad Z. Usmani

**Affiliations:** Myeloma Institute for research & Therapy, University of Arkansas for Medical Sciences, Little Rock, AR

Since the first report of the efficacy of thalidomide in refractory multiple myeloma [1] (MM), there has been remarkable progress made in the biologic understanding of this disease. MM patients have benefited from a surge in biology-based novel drug development based on disease biology with continued improvement in overall survival over the last 13 years [2,3]. But it appears that living longer does not come without a price tag. There are now three randomized phase three clinical trials [4-6], two in transplant eligible patients and one in transplant ineligible patients, which have not only reported on improved progression free survival with lenalidomide (Len), a derivative of thalidomide, maintenance but they also show a higher incidence of second primary malignancies (SPM) with prolonged use of lenalidomide (Table 1). It appears that this effect is more relevant to patients receiving lenalidomide after having received melphalan as part either the transplant regimen or as part of induction in case of transplant ineligible patients. There also appears to be evidence that thalidomide may also potentiate solid SPMs [7], thereby suggesting an immunomodulatory drug (IMiD) class effect associated with alkylator exposure.

**Table 1:** 

Study (Median follow-up)	Treatment Schedule	% SPM
MM-015 (30 months)	MPL-L/MPL Placebo	7% 3%
IFM 2005-02 (45 months)	L Placebo	8% 4%
CALGB 100104 (34 months)	L Placebo	7.8% 2.6%

L=lenalidomide, M=melphalan, P=prednisone

So what does this all mean in the grand scheme of MM therapy in 2012? Although the data identify a small subset of MM patients will likely be affected, SPMs remains a serious issue especially with regard to the myeloid malignancies [8]. We have previously published our experience with myelodysplasia-associated cytogenetic abnormalities [9]. Since autologous stem cell transplantation (ASCT) has been slow to gain acceptance as a standard of care for eligible patients over the last decade, the US centers that have been ASCT proponents are likely to be observing and reporting on the SPM issue before others. For now, the available data speak in favor of Len maintenance in both transplant eligible and ineligible patients. There, however, needs to be an informed discussion with the patients about the potential for SPMs since much is unknown in absence of long-term follow-up on the three randomized studies [4-6].

The Arkansas group is presently studying the baseline whole bone marrow gene-expression profiling, proteomic analyses and single-nucleotide polymorphisms (SNPs) to identify patients who may have the propensity to develop SPMs. This will be most important in the context of low risk (or standard risk) patients who have a longer life expectancy than the high-risk patients [10]. The risk adaptive therapy approach within low risk patients may involve limiting post-transplant IMiD exposure in patients who have a higher potential for SPM development. It may well be that we are looking at the tip of the iceberg in the year 2012 and SPMs may emerge as an important long-term sequela with further follow-up.
